# HIV-related stigma among young men who have sex with men in HIV care in Plateau State Nigeria

**DOI:** 10.3389/fpubh.2025.1473369

**Published:** 2025-01-22

**Authors:** Tolulope O. Afolaranmi, Beth Chaplin, Ayuba I. Zoakah, Phyllis J. Kanki

**Affiliations:** ^1^Department of Community Medicine, University of Jos, Jos, Plateau, Nigeria; ^2^Department of Global Health and Population, Harvard T.H Chan School of Public Health, Boston, MA, United States; ^3^Department of Immunology and Infectious Diseases, Harvard T.H Chan School of Public Health, Boston, MA, United States

**Keywords:** HIV infection, stigma, young men who have sex with men, sexual and gender minority, Nigeria

## Abstract

**Background:**

HIV-related stigma is often expressed as irrational behaviors, negative attitudes, and unfavorable judgments toward people living with or at risk of HIV which remains very common in low- and middle-income countries including Nigeria. This study assessed the level of HIV-related stigma and its associated factors among Young Men who have Sex with Men (YMSM) in HIV care.

**Methodology:**

This was a cross-sectional study conducted among 122 YMSM to assess the level of HIV-related stigma and its associated factors among YMSM in HIV care using respondent driven sampling between July 2023 and April 2024. Quantitative method of data collection was employed and SPSS version 23 was used for data analysis. A *p*-value of ≤ 0.05 was considered statistically significant.

**Results:**

The mean age of the study participants was 22.2 ± 2.0 years with 56 (45.6%) being 22 years and below. The total HIV- related stigma score for the participants was 121.9 ± 18.8 with high-level of stigma reported among 40 (32.8%) of the participants. Significant variation in the mean total HIV-related stigma score was found with duration on HIV treatment with those who had been on treatment for >3 years having a mean score of 117.8 ± 15.2 compared to 110.3 ± 20.1 for those who had been on treatment for <2 years (mean difference: −7.50; 95% Confidence interval: −14.45, −5.51; *p* = 0.035).

**Conclusion:**

This study found a high level of HIV-related stigma among the YMSM in HIV care which is significantly influenced by the duration of time in HIV care.

## Introduction

Stigma and discrimination are human rights and global public health problems which constitute serious impediments to optimal HIV treatment outcomes (1–10). Stigma is a damaging social phenomenon which demeans and devalues individuals or a group of people portraying them as undesirable due to attributes or characteristics that are societally adjudged unacceptable (1, 2, 7, 10–18). HIV-related stigma is often expressed as irrational behaviors, negative attitudes, and unfavorable judgments toward people living with or at risk of HIV which remains very common in low and middle income countries including Nigeria. This can negatively affect the health and wellbeing of people living with HIV (PLWH) by acting as an impediment to optimization of HIV prevention, treatment and support services (19–21). This most often become internalized and leads to undesirable affective, cognitive, and mental health outcomes (2). Internalized HIV-related stigma is the conditional acceptance of negative societal perceptions, characterizations, and labeling of PLWH which can be expressed with self-depreciating feelings such as shame, self-blame, low self-esteem, and self-worth (2, 3). Men who have sex with men (MSM) are disproportionately affected by HIV infection which continues to expand worldwide in both developed and developing countries (7, 21). This group is often stigmatized due to their peculiarities, positions, gender identity and sexual orientation which drives discrimination across sectors of society, including in health care, education, workplace as well as within families and in communities (3, 13, 18). Young men who have sex with men (YMSM) living with HIV face a life-long illness associated with numerous health and social challenges that evolve with time thereby impacting on different areas of their lives. They have to cope with the shock, fear, anger, guilt and shame of having HIV infection as well as the attendant stigmatizing responses associated with their sexual identity which impact on their mental health (22). Studies of HIV-related stigma among YLWHA is of relevance since youth are highly vulnerable to stigma and their numbers are on the rise in society. Youth's vulnerability to HIV-related stigma is exacerbated by social and economic marginalization, as well as the rapid physical and psychosocial transitions (8, 12, 15, 19). However, there is still relatively little empirical evidence on HIV-stigma among people living with HIV particularly for the YMSM in HIV care (20). It was against this backdrop that this study was conducted to assess the level of HIV related stigma and its associated factors among YMSM in HIV care in Plateau State, North central Nigeria as a way of generating geo-specific information and evidence for advocacy and policy change.

## Methodology

### Study area

This study was conducted in Jos, Plateau state, North central Nigeria, which has a population of about 3.2 million people (23). There is a viable MSM networks in the state with functional internal systems and coordination structures including HIV support groups. Additionally, The Society for Family Health (SFH) currently operates One Stop Shop (OSS) for HIV services attending to the HIV care needs of the Key Affected Population in the state (KAP) while the APIN (AIDS Prevention Initiatives in Nigeria) Public Health Initiative is the lead implementing partner supporting PEPFAR funded HIV prevention and care programs in the state.

### Study population

The study population comprised of all YMSM in HIV care in the state affiliated to the HIV support group or within the MSM network in the state who were between 18–24 years of age.

### Study design

This was a cross-sectional study to assess the level of HIV-related stigma and its associated factors among YMSM in HIV care conducted between July 2023 and April 2024 using quantitative method of data collection.

### Sample size estimation

The sample size for this study was determined using the sample size estimation formula for a cross-sectional study (24). Where *n* was the minimum sample size, *Z* is the standard normal deviate at 95% confidence interval (1.96), q is the complementary probability (1 – *p*), d is the precision of the study set at 0.05 and *p* is the prevalence of severe HIV-related stigma among PLHIV from a previous study (6.7%) (6). This gave a minimum sample size of 96.

### Criteria for inclusion in the study

All YMSM in HIV care affiliated to the HIV support group or within the MSM network in the state who were 18–24 years were eligible to participate in the study while those who declined to consent for participation in the study were excluded.

### Sampling technique

Respondent Driven Sampling (RDS) technique was used in this study to recruit consenting eligible YMSM members of MSM community into the study (18, 23, 25). The Respondent-driven sampling (RDS) is a modified form of snowball sampling which offers several advantages for hard to reach groups. A total of 122 YMSM aged 18–24 years currently in HIV care were identified and recruited for the study following informed consent for participation in the study. The facility appointment diary and the HIV case management registers were used to identify the eligible participants in the one- stop shop (OSS) clinic for the key population while the testing peer navigators, some respected YMSM and the key population MSM coordinator facilitated the recruitment of the YMSM accessing HIV care for the state. The recruitment process ended following a 4-week period of saturation when no eligible participants was found.

### Data collection

An interviewer's administration approach to data collection was employed using the Berger's HIV-stigma scale for assessment of HIV related stigma and an adapted tool for demographics as well as social and sexual behaviors of the participants (26, 27). Berger's HIV stigma scale (BHSS) was used for the assessment of perceived HIV stigma among the YMSM in HIV care. Eight trained research assistants who were members of the MSM network carried out the data collection after the completion of the recruitment of the participants into the study. The questionnaires were pretested among MSM in a neighboring state. Prior to the administration of the questionnaire, written informed consent was obtained and documented from all the respondents with the assurance of confidentiality and anonymity of their responses provided.

### Grading of response

The HIV stigma scale consisted of 40 items in four different subscales using a 4-point rating scale of strongly disagree = 1, disagree = 2, agree = 3 and strongly agree = 4 giving a maximum attainable overall score of 160 and minimum attainable score of 40. This scale was further subdivided into four subscales namely; Personalized Stigma subscale (PS) having a total of 18 items with a maximum attainable score of 72 using a 4-point rating scale of strongly disagree = 1, disagree = 2, agree = 3 and strongly agree = 4. The Disclosure Concerns (DC) having 10 items with a maximum attainable score of 40 using a 4-point rating scale of strongly disagree = 1, disagree = 2, agree = 3 and strongly agree = 4. Negative Self-Image (NSI) subscale with a total of 13 items and a maximum attainable score of 52 using a 4 point rating scale also and the Public Attitudes (PA) subscale having 20 items with a maximum attainable score of 80. Furthermore, all 40-items on the scale were cumulatively computed to give the Total HIV Stigma Score (TSS). The scores emanating from the elicited responses are scaled in the positive direction indicating that the higher the scores, the higher the level of HIV-related stigma (6, 8, 10, 28). The Total HIV related stigma score was further categorized into low-level stigma, middle-level stigma and High-level stigma using the percentile graph. Scores between 25th percentile and 50th percentile (40–80) was adjudged to be low-level stigma while scores between 50th percentile and 75th percentile (81–120) as middle-level stigma and those >75th percentile (121–160) categorized as high-level stigma (6, 10, 28).

The explanatory variables in this study were classified as demographic characteristics and sexual behaviors of the respondents. The socio-demographic characteristics included age as at last birthday, religion, highest level of education, sexual orientation categorized as bisexual and homosexual, family type assessed as monogamous and polygamous, family history of same sex orientation assessed as present if the respondents had seen or known any family member engaging in sex with another man, engagement in a paid job assessed as engaged and not engaged, disclosure of HIV status as yes or no, duration on HIV treatment in years and disclosure of sexual orientation as yes or no. The sexual behaviors assessment included type of MSM categorized as penetrative, receptive or both, engagement in anal sex within the last 7days as yes or no, use of condoms in the last anal sex assessed as yes or no. Furthermore, number of male sexual partners within the last 3 months was categorized as 1, 2–5, and 6 or more, self-reported lifetime history of STIs as positive or negative and self-reported engagement in transactional sexual practice within the last 6 months prior to the study which wase assessed as engaged or not engaged. Transactional sex was defined as non-marital, non-commercial sexual relationships motivated by an implicit assumption that sex will be exchanged for material support or other benefits e.g. money (29). The primary outcome variable was total HIV-related stigma score while the secondary outcome variables were the HIV-related stigma subscales scores.

### Data analysis

All filled questionnaires were reviewed for completeness and thereafter serialized. Data analysis was carried out using SPSSS statistical software version 23. Descriptive statistical analysis was carried out on quantitative variables such age of the respondent, age at same sex sexual debut, total HIV-related stigma scores and the scores of stigma subscales. These quantitative variables were expressed with mean and standard deviation as their summary indices after fulfilling the assumptions of normality while duration on HIV treatment was found to be skewed and median and interquartile range were employed as its summary indices. Other explanatory variables such as age group, marital status, sexual orientation, family history of same sex orientation, and grading of the HIV stigma etc were presented in frequency table expressed in frequencies and percentages. Correlation between the Total HIV-related stigma scores and the scores of the subscales was established using Pearson's correlation. The correlation coefficient (*r*) for PS subscale was 0.91, DC subscale was 0.75, NSI subscale 0.90 and PA subscale 0.98. The variation in mean total HIV-related stigma score was tested using unpaired students *T*-test for explanatory variables expressed in two categories while one way analysis of variance was used for those expressed in three or more categories. Mean difference and 95% confidence interval were used as point and interval estimates of the difference in mean scores in the student *T*-test with a probability value of < 0.05 was considered statistically significant.

## Results

The mean age of the study participants was 22.2 ± 2.0 years with 56 (45.6%) being 22 years and below. Fifty-two (42.6%) of the respondents identified as bisexual and 66 (54.1%) debuted sex with a male partner below 16 years of age. Fifty-one (41.8%) of the study participants had completed tertiary education while 73 (59.3%) of them had disclosed their HIV status to at least one person in the past (Table 1).

**Table 1:** Sociodemographic characteristics of the participants.

**Characteristics**	**Frequency (*n* = 122)**	**Percentage**
**Age group (years)**		
≤ 22	56	45.9
23 and above	66	54.1
Mean age ± SD	22.2 ±2.0 years	
**Religion**		
Christianity	50	41.0
Islam	72	59.0
**Sexual orientation/identity**		
Bisexual	52	42.6
Homosexual	70	57.4
**Age at same sex sexual debut (year)**		
≤ 16	66	54.1
17 and above	56	44.9
Mean age ± SD	15.6 ± 3.0 years	
**Family of origin type**		
Monogamous	67	54.9
Polygamous	55	45.1
**Family history of same sex practice**		
Present	25	20.5
Absent	97	79.5
**Highest level of education**		
None	1	0.8
Primary	6	4.9
Secondary	64	52.5
Tertiary	51	41.8
**Employment in paid job**		
Yes	46	37.7
No	76	62.3
**Place of residence**		
Urban	86	70.5
Rural	36	29.5
**Disclosure of HIV status**		
Yes	73	59.8
No	49	40.2
**Duration on HIV treatment (years)**		
≤ 2	79	64.8
3 and above	43	35.2
Median (IOR)	2 (1–4) years	
**Disclosure of sexual orientation**		
Yes	59	48.4
No	63	51.6

SD, standard deviation; IQR, interquartile range.

With regards to the sexual behaviors of the participants, 31 (25.4%) were penetrative MSM, 18 (14.8%) receptive and 73 (59.8%) both receptive and penetrative in their anal sex sexual preferences. Engagement in transactional sex was self-reported by 41 (33.6%) with 71 (58.2%) reported engagement in anal sex within the last 7 days of the assessment and 33 (46.5%) of those who had engaged in anal sex did not use condoms (Table 2).

**Table 2:** Sexual behaviors of the participants.

**Characteristics**	**Frequency (*n* = 122)**	**Percentage**
**Category of MSM**		
Penetrative	31	25.4
Receptive	18	14.8
Both	73	59.8
**Number of male sexual partners in the last 3 months**		
1	28	23.0
2–5	78	63.9
6 and more	16	13.1
Median (IOR)	2 (2–4)	
**Engagement in transactional sex**		
Yes	41	33.6
No	81	66.4
**Engagement in anal sex in the last 7 days**		
Yes	71	58.2
No	52	41.8
Condom use in the last anal sex^*^(***n*** = **71)**		
Yes	38	53.5
No	33	46.5
**Lifetime history of STIs apart from HIV**		
Positive	41	33.6
Negative	81	66.4

^*^Subset of engagement in anal sex within the last 7 days.

IQR, interquartile range.

In the assessment of the HIV related stigma, the total HIV-related stigma score for the participants was 121.9 ± 18.8 with 40 (32.8%) being classified as high-level stigma and 75 (61.5%) with middle-level stigma. The mean personalized stigma subscale score of 48.3 ± 11.7 was reported among the participants while average score for stigma associated with disclosure subscale was found to be 30.9 ± 4.2. Mean scores for negative self-image and public attitude stigma subscales were 35.8 ± 6.6 and 57.2 ± 10.4 respectively (Table 3).

**Table 3:** HIV related stigma assessment scores of the participants.

**Stigma scale score**	**Mean ±SD**	**Minimum attainable score**	**Maximum attainable score**
Personalized stigma subscale	48.3 ± 11.7	18.0	72.0
Disclosure concern subscale	30.9 ± 4.2	10.0	40.0
Negative self-image subscale	35.8 ± 6.6	13.0	52.0
Public attitude subscale	57.2 ± 10.4	20.0	80.0
Total HIV stigma scale	121.9 ± 18.8	40.0	160.0
Grading of total HIV stigma	Freq (%)		
Low-level stigma	7 (5.7)		
Middle-level stigma	75 (61.5)		
High-level stigma	40 (32.8)		
Total	122 (100.0)		

SD, standard deviation.

The variation in the mean overall HIV-related stigma scores across the demographic characteristics and the sexual behaviors of the respondents is shown in Table 4. In this study, age, sexual orientation, age at same sex debut, level of education, employment status, disclosure of sexual orientation and engagement in transactional sex among others did not show any statistically significant variation in the mean total HIV-related sigma scores. However, significant variation in mean total HIV-related stigma score was found with duration on HIV treatment as those who had been on treatment for 3 years and more having a mean score of 117.8 ± 15.2 compared to 110.3 ± 20.1 among those who had been on treatment for 2 years of less (mean difference: −7.50; 95% Confidence interval: −14.45, −5.51; *p* = 0.035; Table 4). Furthermore, no statistically significant variation was found in the assessment of the mean scores of the personalized stigma and public attitudes subscales across the demographic characteristics and sexual behaviors of the respondents. However, duration on HIV treatment showed statistically significant difference on the disclosure concern subscale with those who had been on treatment for 3 years and more having a mean score of 32.4 ± 3.5 compared to 30.2 ± 4.3 of those who had been on treatment for 2 years of less (mean difference: −2.22; 95% Confidence interval: −3.74, −0.70; *p* = 0.004). Similarly, respondents who had been on treatment for 3 years and more had a higher mean score of 37.5 ± 4.9 compared to 34.9 ± 7.2 for those who had been on treatment for 2 years of less (mean difference: −2.64; 95% Confidence interval: −5.08, −0.19; *p* = 0.035) on the negative self-image subscale (Tables 5–8).

**Table 4:** Variation in mean Total HIV stigma score by characteristics of the participants.

**Characteristics**	***n***	**Mean ±SD**	**Mean diff**	**95% Conf. inter**.	***T*-test**	***p*-value**
**Age group (years)**						
≤ 22	56	113.3 ± 17.9	0.63	−6.16, 7.42	0.183	0.855
23 and above	66	112.6 ± 19.6				
**Sexual orientation**						
Bisexual	52	110.5 ± 17.4	−4.17	−10.97, 2.63	−1.213	0.227
Homosexual	70	114.7 ± 19.7				
**Age at same sex sexual debut (year)**						
≤ 16	66	112.4 ± 19.1	−1.19	−7.98, 5.60	−0.347	0.729
17 and above	56	113.6 ± 18.6				
**Family of origin type**						
Monogamous	68	114.3 ± 19.2	3.23	−3.56, 10.01	0.942	0.348
Polygamous	54	111.7 ± 18.3				
**Family history of same sex practice**						
Present	25	110.9 ± 21.0	−2.31	−10.83, 6.21	0.537	0.592
Absent	97	113.2 ± 18.3				
**Highest level of education**						
None	1	135.0 ± 0.0	–	–	1.221^*^	0.305
Primary	6	122.7 ± 11.8				
Secondary	64	113.3 ± 17.5				
Tertiary	51	110.8 ± 20.7				
**Employment in paid job**						
Yes	46	111.1 ± 16.3	−2.90	−9.82, 4.10	−0.812	0.418
No	76	114.0 ± 20.2				
**Place of residence**						
Urban	86	113.4 ± 19.0	2.10–5.17,	9.64	0.598	0.551
Rural	36	111.3 ± 18.6				
**Duration on HIV treatment (years)**						
≤ 2	79	110.3 ± 20.1	−7.50	−14.45, −5.51	−2.137	0.035
3 and above	43	117.8 ± 15.2				
**Disclosure of HIV status**						
Yes	73	111.8 ± 18.3	−2.85	−9.73, 4.04	−0.812	0.415
No	49	114.6 ± 19.5				
**Category of MSM**						
Penetrative	31	112.8 ± 17.9	–	–	0.111^*^	0.895
Receptive	18	114.8 ± 13.7				
Both	73	112.5 ± 20.4				
**Number of male sexual partners in the last 3 months**						
1	28	112.0 ± 19.0	–	–	0.713^*^	0.492
2–5	78	114.2 ± 17.7				
6 and more	16	108.2 ± 23.7				
**Engagement in transactional sex**						
Yes	41	110.9 ± 19.2	−3.00	−10.17, 4.12	−0.838	0.404
No	81	113.9 ± 18.6				
**Condom use in the last anal sex**						
Yes	82	112.0 ± 18.9	−2.80	−9.96, 4.41	−0.647	0.446
No	40	114.8 ± 18.7				
**Lifetime history of STIs apart from HIV**						
Positive	41	113.8 ± 19.8	1.40	−5.77, 8.54	0.383	0.702
Negative	81	112.4 ± 18.4				
**Disclosure of sexual orientation**						
Yes	59	110.9 ± 19.2	−3.90	−10.60, 2.87	−1.136	0.258
No	63	114.8 ± 18.4				

SD, standard deviation.

^*^F-test, T-test = unpaired T-test.

**Table 5:** Variation in mean personalized stigma subscale score by characteristics of the participants.

**Characteristics**	***n***	**Mean ±SD**	**Mean diff**	**95% Conf. inter**.	***T*-test**	***p*-value**
**Age group (years)**						
≤ 22	56	48.4 ± 11.3	0.14	−4.08, 4.36	0.809	0.948
23 and above	66	48.3 ± 12.1				
**Sexual orientation**						
Bisexual	52	46.6 ± 10.7	−3.07	−7.28, 1.15	−1.441	0.152
Homosexual	70	49.6 ± 12.3				
**Age at same sex sexual debut (year)**						
≤ 16	66	48.2 ± 7.3	−0.40	−4.62, 3.52	−0.189	0.851
17 and above	56	48.6 ± 11.7				
**Family of origin type**						
Monogamous	68	48.8 ± 11.9	1.07	−3.16, 5.30	0.500	0.618
Polygamous	54	47.7 ± 11.4				
**Family history of same sex practice**						
Present	25	48.5 ± 12.3	0.28	−5.01, 5.57	0.106	0.916
Absent	97	48.2 ± 11.6				
**Highest level of education**						
None	1	61.0 ± 0.0	–	–	0.973^*^	0.408
Primary	6	54.0 ± 10.8				
Secondary	64	48.4 ± 11.7				
Tertiary	51	47.4 ± 11.8				
**Employment in paid job**						
Yes	46	46.4 ± 10.7	−3.09	−7.39, 1.21	−1.421	0.158
No	76	49.5 ± 12.1				
**Place of residence**						
Urban	86	48.7 ± 12.0	1.34	−3.26, 5.95	0.578	0.564
Rural	36	47.4 ± 11.1				
**Duration on HIV treatment (years)**						
≤ 2	79	47.3 ± 12.1	−2.97	−7.33, −1.40	−1.344	0.181
3 and above	43	50.3 ± 10.7				
**Disclosure of HIV status**						
Yes	73	47.6 ± 10.3	−1.76	−6.03, 2.52	−0.814	0.417
No	49	49.4 ± 13.0				
**Category of MSM**						
Penetrative	31	48.2 ± 11.6	–	–	0.040^*^	0.961
Receptive	18	49.1 ± 9.7				
Both	73	48.2 ± 12.3				
**Number of male sexual partners in the last 3 months**						
1	28	47.9 ± 11.2	–	–	0.232^*^	0.713
2–5	78	48.8 ± 11.5				
6 and more	16	46.8 ± 13.				
**Engagement in transactional sex**						
Yes	41	46.5 ± 11.9	−2.71	−7.13, 1.71	−1.213	0.227
No	81	49.3 ± 11.6				
**Condom use in the last anal sex**						
Yes	82	47.9 ± 11.4	−1.25	−5.72, 3.22	−0.552	0.582
No	40	49.2 ± 12.3				
**Lifetime history of STIs apart from HIV**						
Positive	41	48.4 ± 11.7	0.10	−4.41, 4.50	0.020	0.984
Negative	81	48.3 ± 11.7				
**Disclosure of sexual orientation**						
Yes	59	47.6 ± 11.6	−1.50	−5.70, 2.69	0.710	0.497
No	63	49.1 ± 11.8				

SD, standard deviation.

^*^F-test, T-test= unpaired T-test.

**Table 6:** Variation in mean disclosure concern subscale score by characteristics of the participants.

**Characteristics**	***n***	**Mean ±SD**	**Mean diff**	**95% Conf. inter**.	***T*-test**	***p*-value**
**Age group (years)**						
≤ 22	56	30.9 ± 3.7	−0.14	−1.65, 1.36	−0.188	0.851
23 and above	66	31.0 ± 4.5				
**Sexual orientation**						
Bisexual	52	31.0 ± 4.3	0.11	−1.40, 1.63	0.149	0.882
Homosexual	70	30.9 ± 4.1				
**Age at same sex sexual debut (year)**						
≤ 16	66	30.6 ± 4.2	−0.85	−2.35, 0.65	−1.12	0.265
17 and above	56	31.4 ± 4.1				
**Family of origin type**						
Monogamous	68	31.1 ± 4.2	0.35	−1.16, 1.86	0.456	0.649
Polygamous	54	30.7 ± 4.2				
**Family history of same sex practice**						
Present	25	30.1 ± 5.2	−0.10	−2.89, 0.87	−1.048	0.297
Absent	97	31.1 ± 3.9				
**Highest level of education**						
None	1	32.0 ± 0.0	–	–	0.120^*^	0.948
Primary	6	31.8 ± 2.5				
Secondary	64	30.9 ± 5.6				
Tertiary	51	30.9 ± 4.2				
**Employment in paid job**						
Yes	46	30.3 ± 3.6	0.56	−0.99, 2.10	0.716	0.475
No	76	30.7 ± 4.5				
**Place of residence**						
Urban	86	31.0 ± 4.3	0.30	−1.34, 1.95	0.363	0.718
Rural	36	30.7 ± 4.0				
**Duration on HIV treatment (years)**						
≤ 2	79	30.2 ± 4.3	−2.22	−3.74, −0.70	−2.870	0.004
3 and above	43	32.4 ± 3.5				
**Disclosure of HIV status**						
Yes	73	30.9 ± 4.3	−0.01	−1.54, 1.52	−0.009	0.993
No	49	30.9 ± 4.0				
**Category of MSM**						
Penetrative	31	30.7 ± 4.5	–	–	0.547^*^	0.580
Receptive	18	30.2 ± 3.2				
Both	73	31.2 ± 4.2				
**Number of male sexual partners in the last 3 months**						
1	28	31.0 ± 4.8	–	–	1.235^*^	0.295
2–5	78	31.2 ± 3.7				
6 and more	16	29.5 ± 4.9				
**Engagement in transactional sex**						
Yes	41	30.7 ± 4.4	−0.42	−0.20, 1.17	−0.519	0.605
No	81	31.1 ± 4.1				
**Condom use in the last anal sex**						
Yes	82	31.0 ± 4.5	0.13	−1.47, 1.72	0.156	0.877
No	40	30.9 ± 3.5				
**Lifetime history of STIs apart from HIV**						
Positive	41	31.3 ± 4.7	0.61	−0.97, 2.20	0.766	0.445
Negative	81	30.7 ± 4.0				
**Disclosure of sexual orientation**						
Yes	59	30.6 ± 4.4	−0.60	−2.09, 0.90	−0.787	0.433
No	63	31.2 ± 3.9				

SD, standard deviation.

^*^F-test, T-test= unpaired T-test.

**Table 7:** Variation in mean negative self-image subscale score by characteristics of the participants.

**Characteristics**	***n***	**Mean ±SD**	**Mean diff**	**95% Conf. inter**.	***T*-test**	***p*-value**
**Age group (years)**						
≤ 22	56	36.0 ± 7.0	0.35	−2.04, 2.74	0.291	0.772
23 and above	66	35.7 ± 6.4				
**Sexual orientation**						
Bisexual	52	34.9 ± 6.6	−0.16	−3.97, 0.82	−1.304	0.195
Homosexual	70	36.5 ± 6.7				
**Age at same sex sexual debut (year)**						
≤ 16	66	35.9 ± 6.6	0.01	−2.35, 2.44	0.037	0.970
17 and above	56	35.8 ± 6.7				
**Family of origin type**						
Monogamous	68	36.5 ± 6.5	1.62	−0.77, 4.00	1.344	0.181
Polygamous	54	34.9 ± 6.7				
**Family history of same sex practice**						
Present	25	35.0 ± 6.8	−1.00	−3.97, 2.03	−0.640	0.524
Absent	97	36.0 ± 6.6				
**Highest level of education**						
None	1	46.0 ± 0.0	–	–	1.756^*^	0.159
Primary	6	39.8 ± 2.9				
Secondary	64	35.9 ± 5.9				
Tertiary	51	35.1 ± 7.5				
**Employment in paid job**						
Yes	46	35.2 ± 5.6	−1.00	−3.43, 1.47	−0.791	0.431
No	76	36.2 ± 7.2				
**Place of residence**						
Urban	86	36.0 ± 6.5	0.50	−2.19, 3.04	0.322	0.748
Rural	36	35.5 ± 7.1				
**Duration on HIV treatment (years)**						
≤ 2	79	34.9 ± 7.2	−2.64	−5.08, −0.19	−2.130	0.035
3 and above	43	37.5 ± 4.9				
**Disclosure of HIV status**						
Yes	73	35.3 ± 6.7	−1.28	−3.70, 1.15	−1.044	0.299
No	49	36.6 ± 6.5				
**Category of MSM**						
Penetrative	31	35.6 ± 6.0	–	–	0.300^*^	0.741
Receptive	18	36.9 ± 5.8				
Both	73	36.7 ± 7.1				
**Number of male sexual partners in the last 3 months**						
1	28	35.1 ± 6.5	–	–	0.571^*^	0.567
2–5	78	36.3 ± 6.1				
6 and more	16	34.8 ± 9.4				
**Engagement in transactional sex**						
Yes	41	35.8 ± 6.9	−0.10	−2.60, 2.45	−0.056	0.955
No	81	35.9 ± 6.6				
**Condom use in the last anal sex**						
Yes	82	35.3 ± 6.6	−1.70	−4.23, 0.82	−1.340	0.183
No	40	37.0 ± 6.6				
**Lifetime history of STIs apart from HIV**						
Positive	41	36.6 ± 7.0	1.10	−1.41, 3.62	0.869	0.387
Negative	81	35.5 ± 6.5				
**Disclosure of sexual orientation**						
Yes	59	35.0 ± 6.8	−1.60	−4.00, 0.73	−1.368	0.174
No	63	36.6 ± 6.4				

SD, standard deviation.

^*^F-test, T-test= unpaired T-test.

**Table 8:** Variation in mean Public attitude subscale score by characteristics of the participants.

**Characteristics**	***n***	**Mean ±SD**	**Mean diff**	**95% Conf. inter**.	***T*-test**	***p*-value**
**Age group (years)**						
≤ 22	56	57.0 ± 9.5	−0.29	−4.06, 3.48	−0.151	0.880
23 and above	66	57.3 ± 11.2				
**Sexual orientation**						
Bisexual	52	55.9 ± 9.2	−2.20	−5.93, 1.63	−1.126	0.262
Homosexual	70	58.1 ± 11.2				
**Age at same sex sexual debut (year)**						
≤ 16	66	56.8 ± 10.7	−0.70	−4.47, 3.06	−0.369	0.713
17 and above	56	57.5 ± 10.1				
**Family of origin type**						
Monogamous	68	57.9 ± 10.6	1.78	−1.99, 5.54	0.933	0.353
Polygamous	54	56.2 ± 10.2				
**Family history of same sex practice**						
Present	25	55.9 ± 12.6	−1.49	−6.21, 3.23	−0.623	0.534
Absent	97	57.4 ± 9.9				
**Highest level of education**						
None	1	71.0 ± 0.0	–	–	1.471^*^	0.226
Primary	6	62.7 ± 4.9				
Secondary	64	57.5 ± 9.5				
Tertiary	51	55.8 ± 11.7				
**Employment in paid job**						
Yes	46	56.1 ± 9.0	−1.72	−5.58, 2.15	−0.880	0.381
No	76	57.8 ± 11.2				
**Place of residence**						
Urban	86	57.5 ± 10.8	1.13	−2.99, 5.24	0.543	0.588
Rural	36	56.4 ± 9.7				
**Duration on HIV treatment (years)**						
≤ 2	79	55.9 ± 11.2	−3.71	−7.58, −0.16	−1.897	0.060
3 and above	43	59.6 ± 8.6				
**Disclosure of HIV status**						
Yes	73	56.6 ± 10.1	−1.38	−5.20, 2.46	−0.713	0.477
No	49	57.9 ± 10.9				
**Category of MSM**						
Penetrative	31	57.0 ± 10.1	–	–	0.121^*^	0.886
Receptive	18	58.3 ± 6.8				
Both	73	56.9 ± 11.4				
**Number of male sexual partners in the last 3 months**						
1	28	57.5 ± 10.7	–	–	1.024^*^	0.362
2–5	78	57.7 ± 9.6				
6 and more	16	53.7 ± 13.5				
**Engagement in transactional sex**						
Yes	41	55.6 ± 10.9	−2.40	−6.35, 1.55	−1.204	0.231
No	81	58.0 ± 6.6				
**Condom use in the last anal sex**						
Yes	82	55.7 ± 10.6	−1.41	−5.40, 2.59	−0.697	0.487
No	40	58.1 ± 10.1				
**Lifetime history of STIs apart from HIV**						
Positive	41	57.4 ± 11.1	0.40	−3.62, 4.33	0.176	0.861
Negative	81	57.0 ± 10.2				
**Disclosure of sexual orientation**						
Yes	59	56.1 ± 10.8	−2.04	−5.78, 1.70	−1.081	0.282
No	63	58.1 ± 10.1				

SD, standard deviation.

^*^= F test, T-test= unpaired T-test.

## Discussion

HIV-related stigma poses to be an imminent clog in the cascade of HIV treatment and continuum of care thereby potentiating poor adherence, disclosure refusal, self-blame, depression, isolation and treatment failure (6, 30–32). In this study, high level of HIV-related stigma was reported among the YMSM in HIV care as demonstrated by high total HIV stigma scores and also across all the subscales of personalized stigma, disclosure, negative self-image and public attitude. The high total HIV-related stigma scores found in this study are consistent with findings of other studies conducted in India, China and United States of America (7, 18, 20, 33, 34). However, other studies conducted in United States of America reported slightly lower levels of HIV-related stigma in comparison to this study (1, 19). The similarities observed with the findings of this study and others cited ones could be attributable to the use of similar stigma assessment tools, study design and MSM in HIV care as the study participants. However, adults in HIV care who were not members of the MSM community were assessed for HIV related stigma in some of the other referenced studies. It is important to state that the MSM population and particularly YMSM are a vulnerable group perceived to be exhibiting behaviors and sexual identities that contradict social norms, cultures and traditions in most climes (18). This in itself has been the foremost driver of HIV-related stigma among the MSM population and significant contributor to the relegation of this group to the closet. The combination stigmatization and repressive legislations pose serious challenges to HIV care and if not addressed, it will continue to negatively impact the desired HIV/AIDS treatment outcomes and possibly contribute to reversing the gains already achieved in the HIV treatment landscape.

Total HIV-related stigma score was found to increase with duration in HIV care in this study, however, other studies reported abandonment by spouse, isolation by family, exclusion from social activities, bisexual identity, bartering sex, injection drug use, disclosure and depression as factors driving HIV-related stigma (6, 18, 20). It is therefore imperative that development or deployment of innovative and context specify strategies for stigma reduction in HIV care is essential but more importantly such strategies should target the peculiarities of the YMSM in HIV care. The cross-sectional design of this study will not support the establishment of temporal relationship particularly between duration in HIV care and the total HIV-related stigma score which was found to be statistically significant. Additionally, the tool used for the assessment of HIV related stigma focused on perceived stigma making it important that further studies be conducted to examine the lived experience of stigma in this population and its potential environmental drivers as nexus for generating empirical evidence in providing the needed psychological, psychosocial and social support necessary to mitigate the impact of stigma on the YMSM living with HIV.

## Conclusion

This study found a high level of HIV- related stigma among the Young Men who Have Sex with Men in HIV care which is significantly influenced by the duration of time in HIV care. The study also found high levels of sub-scales of stigma such as Personalized Stigma, Disclosure Concerns, Negative Self-Image and Public Attitudes.

## Data Availability

The raw data supporting the conclusions of this article will be made available by the authors, without undue reservation.
